# Predicting hosts based on early SARS-CoV-2 samples and analyzing the 2020 pandemic

**DOI:** 10.1038/s41598-021-96903-6

**Published:** 2021-08-31

**Authors:** Qian Guo, Mo Li, Chunhui Wang, Jinyuan Guo, Xiaoqing Jiang, Jie Tan, Shufang Wu, Peihong Wang, Tingting Xiao, Man Zhou, Zhencheng Fang, Yonghong Xiao, Huaiqiu Zhu

**Affiliations:** 1 State Key Laboratory for Turbulence and Complex Systems, Department of Biomedical Engineering, College of Engineering, Peking University, Beijing, 100871 China; 2 Center for Quantitative Biology, Peking University, Beijing, 100871 China; 3 Department of Biomedical Engineering, Georgia Institute of Technology and Emory University, Atlanta, GA 30332 USA; 4 Peking University-Tsinghua University-National Institute of Biological Sciences (PTN) Joint PhD Program, School of Life Sciences, Peking University, Beijing, 100871 China; 5 Institute of Medical Technology, Peking University Health Science Center, Beijing, 100191 China; 6 State Key Laboratory for Diagnosis and Treatment of Infectious Diseases, National Clinical Research Center for Infectious Diseases, Collaborative Innovation Center for Diagnosis and Treatment of Infectious Diseases, The First Affiliated Hospital, College of Medicine, Zhejiang University, Hangzhou, 310006 China

**Keywords:** Virology, Computational biology and bioinformatics, Computational models

## Abstract

The SARS-CoV-2 pandemic has raised concerns in the identification of the hosts of the virus since the early stages of the outbreak. To address this problem, we proposed a deep learning method, DeepHoF, based on extracting viral genomic features automatically, to predict the host likelihood scores on five host types, including plant, germ, invertebrate, non-human vertebrate and human, for novel viruses. DeepHoF made up for the lack of an accurate tool, reaching a satisfactory AUC of 0.975 in the five-classification, and could make a reliable prediction for the novel viruses without close neighbors in phylogeny. Additionally, to fill the gap in the efficient inference of host species for SARS-CoV-2 using existing tools, we conducted a deep analysis on the host likelihood profile calculated by DeepHoF. Using the isolates sequenced in the earliest stage of the COVID-19 pandemic, we inferred that minks, bats, dogs and cats were potential hosts of SARS-CoV-2, while minks might be one of the most noteworthy hosts. Several genes of SARS-CoV-2 demonstrated their significance in determining the host range. Furthermore, a large-scale genome analysis, based on DeepHoF’s computation for the later pandemic in 2020, disclosed the uniformity of host range among SARS-CoV-2 samples and the strong association of SARS-CoV-2 between humans and minks.

## Introduction

The global COVID-19 pandemic caused by severe acute respiratory syndrome coronavirus 2 (SARS-CoV-2) has created a long-lasting quest to look for hosts of the virus since the pandemic outbreak; meanwhile, the majority view is that the virus probably originated from bats^1^. To date, there have been many discussions on the potential hosts despite the initially suspected host, the pangolin (Manis javanica)^2,3^. Most of the suppositions were based on the increasing cases of animal infection, such as dogs, cats, tigers, lions, and minks^4,5^. Several studies have performed experiments to investigate the susceptibility of a limited number of model animals^6–8^. At the same time, some studies have attempted to reveal the range of hosts based on analyses using molecular sequences or structural information^9,10^. For instance, Damas et al.^10^ conducted a computational analysis based on host receptor similarities using the angiotensin-converting enzyme 2 (ACE2) protein and evaluated the infection risks for a broad range of animals. As the pandemic spreads, minks, which were not referred to as a high probability to be host animals in the above peer-review articles, have been frequently reported to be largely infected with COVID-19 over the world^5^, and were the only known animal reported to transmit SARS-CoV-2 to humans^11,12^. It is worth mentioning that in January 2020, we reported in the form of a preprint archive predicting minks as a potential host based on the six earliest sequenced SARS-CoV-2 isolates^13^. However, the impact of the current pandemic prompts people to review the issue of host determination for SARS-CoV-2. This raises a new challenge, which is how to implement and improve the capability of computational methods to predict the hosts of a novel virus such as SARS-CoV-2, especially when we have a relatively small number of samples of sequencing viral data at the early stage of the pandemic outbreak. It is certainly constructive for a similar pandemic caused by novel viruses in the future.

Generally, the host range of viruses is dependent on the molecular interactions between viruses and host cells including receptor recognition, adaption to the host cellular machinery and the evasion of the innate immune recognition^14^. Of these, receptor recognition that facilitates the attachment of viruses to host cells is the primary step. Thus, the glycoproteins that viruses use to recognize the host receptor as well as whole genome sequences are widely used in identifying the potential hosts of viruses^1^. To detect the potential host and pathogenicity of novel viruses, the conventional computational methods are almost based on the similarity among either the viral genomic compositions or the host receptors. Both strategies lie in the assumption that phylogenetic relatedness reflects host associations. Even though the related viruses often have closely related hosts, Babayan et al. found that the algorithm, which predicted host associations from viral phylogenetic relatedness, could only accurately identify the reservoir hosts of 58.1 ± 0.07% (standard deviation) of viruses^15^. Additionally, the similarity-based methods based on similarity are constrained by the reference sequences available in the databases.

Until now, several published tools aiming to identify the hosts of viruses exceeded the limitation of sequence-similarity-based strategies by machine learning methods with viral sequences or their genomic traits related to virus-host interactions, such as ViralHostPredictor^15^, HostPhinder^16^, WIsH^17^, Host Taxon Predictor^18^, and VIDHOP^19^. While these tools performed well under some conditions, they are actually not considered feasible to be applied to a novel virus without knowledge of host range, like SARS-CoV-2. HostPhinder and WIsH predict the hosts for bacteriophages only and they are inappropriate for non-phage viruses. Host Taxon Predictor focuses on distinguishing bacteriophages and eukaryotic viruses. ViralHostPredictor predicts hosts and the existence and identity of arthropod vectors for human-infective RNA viruses by gradient boosting machines with the features of selected evolutionary genomic traits and phylogenetic information. It also illustrated the better ability of machine learning methods to predict viral hosts compared to sequence similarity-based methods. However, ViralHostPredictor cannot determine whether the human is the host of a novel virus. With the utilization of evolutionary signatures, ViralHostPredictor lacks the power to predict incidental hosts which do not maintain a long-term circulation of new viruses. Moreover, the predictive abilities of the methods above rely on handcrafted features like codon pair scores, k-mer frequencies and amino acid biases, which might neglect other important information encoded within the virus genomes. VIDHOP, a deep-learning-based tool, is designed to predict potential hosts of viruses, but its application was limited to three viral species: influenza A, rabies lyssavirus and rotavirus A.

To address the challenge of predicting probable hosts of novel viruses, especially those without close neighbors in phylogeny, we proposed the host prediction algorithm DeepHoF (Deep learning-based Host Finder) in the current study. Developed based on the BiPath Convolutional Neural Network (BiPathCNN), DeepHoF automatically extracts the genomic features from the input viral sequences. The model finally outputs five host likelihood scores and their *p* values on five host types, including plant, germ, invertebrate, non-human vertebrate (referring to vertebrates other than humans) and human, where all the living organism hosts are covered. DeepHoF was designed as a five-class classifier containing five independent nodes in the output layer with sigmoid activation and binary cross-entropy loss function for each node, corresponding to five independent binary classifications on the five host types individually. DeepHoF made up for the lack of an efficient method applicable for any novel virus and significantly outperformed the Basic Local Alignment Search Tool (BLAST)-based strategy with an evidently high AUC of 0.975 on the classification of the five host types. We predicted and analyzed the 17 earliest sampled SARS-CoV-2 isolates, which provided essential information regarding the early phases of the epidemic of the virus. In January 2020, we reported host prediction at the earliest stage of the outbreak using the isolates available in December 2019^13^. DeepHoF evaluated the host likelihood scores on humans and non-human vertebrates for the earliest samples and characterized the isolates with their host likelihood score profiles. As there is a gap in the inference of host species for SARS-CoV-2 using the tools which were state of the art, we conducted a deep analysis on the host likelihood score profiles predicted by DeepHoF to find the likely hosts, including both reservoirs and susceptible hosts which are not discriminated in this study. We inferred that minks, bats, dogs and cats were probable hosts, while minks may be one of the most noteworthy hosts. This inference was supported by the known infection data or animal experiments during the pandemic. Based on our model, several genes of SARS-CoV-2 were further investigated and demonstrated their significance in determining the host likelihood scores on human or the host range for SARS-CoV-2, respectively. With a large-scale genome analysis based on DeepHoF’s computation for the later pandemic, the uniformity of host inference among a large number of SARS-CoV-2 samples was verified, and the association of SARS-CoV-2 between humans and minks was disclosed. Supported by the satisfactory performance on five host type classifications and the successful application in SARS-CoV-2, DeepHoF has the capability to provide reliable host information of novel viruses and is expected to narrow the time lag between the discovery of a novel virus and prevention at the early stage of an epidemic.

## Results

### Performance of the DeepHoF algorithm

**Figure 1:**
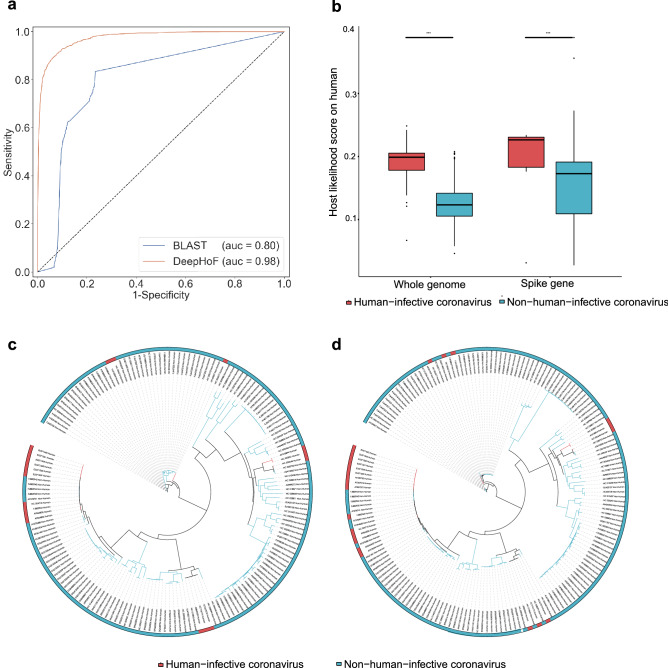
DeepHoF outperforms BLAST. (**a**) Average ROC curves and AUC values of DeepHoF and BLAST. DeepHoF performs better than BLAST on average AUC of five host types. (**b**) Comparison of human host likelihood scores predicted by DeepHoF between human-infecting and non-human-infecting coronaviruses on human. The former performed higher probabilities than the latter (two-sided unpaired Welch Two Sample *t* test, *t*_(43.843)_ = 8.265 and *t*_(38.016)_ = 4.674, *p* values = 1.732 × 10^–10^ and 3.657 × 10^–5^. ***: *p* value $<$ 0.0001, *t* values and degrees of freedom were presented as *t*_(df)_). (**c**,**d**) Phylogenetic analyses of whole genomes and S genes of coronaviruses respectively. Maximum-likelihood phylogenic trees were built by RAxML^39^ with 1000 bootstrap replicates and visualized with iTOL^41^. There were clear non-human-infecting gaps between closely related human-infecting coronaviruses in the phylogenetic trees for either whole genomes or S genes. For some human-infective viruses, their closest neighbors in phylogeny were non-human-infective ones (red: human-infective coronaviruses; blue: non-human-infective coronaviruses).

The DeepHoF algorithm is designed as a five-class classifier using the deep learning method of BiPathCNN (see “Materials and methods”). Herein five likelihood scores on five host types, including plants, germs, invertebrates, non-human vertebrates, and humans, were calculated by DeepHoF. Each host likelihood score is the confidence for the prediction of infecting the corresponding host type, rather than the probability value in statistics. The host likelihood score profile consisting of five predicted scores, was then analysed in depth to find the specific predicted hosts of a novel virus such as SARS-CoV-2 in this study. As mentioned above, the existing bioinformatics tools^15–19^ were not designed to predict the host likelihood scores on the five host types for any given virus, and thus cannot be compared with DeepHoF directly. Therefore, we compared the performance of the DeepHoF model with BLAST (details of finding the host using BLAST are described in Supplementary Methods), adopting six classification metrics: true-positive rate (TPR), false-positive rate (FPR), area under the curve (AUC), precision, accuracy and F1-score. To assess the performance of predicting novel viruses, we used training and test datasets divided in chronological order^20^ (“Materials and methods”). There was no overlap of virus species in the training and test sets. With a higher AUC of 0.975, DeepHoF can significantly outperform BLAST (with an average AUC of 0.800) as shown in Fig. 1a and Table 1 (a detailed comparison on each host type is shown in Supplementary Fig. S1; Table S1).

**Table 1:** Performance metrics of DeepHoF and BLAST.

Methods	Precision	Accuracy	TPR	FPR	AUC	F1-score
BLAST	0.692	0.895	0.894	0.105	0.800	0.899
DeepHoF	0.967	0.967	0.835	0.007	0.975	0.958

*TPR* true-positive rate, *FPR* false-positive rate, *AUC* area under the curve.

In addition, we analysed the human-infective and non-human-infective coronaviruses using their whole genome sequences via DeepHoF and the phylogenetic method. As shown in Fig. 1b (the left), DeepHoF could identify evidently higher probabilities of human-infective coronaviruses to infect humans (two-sided unpaired Welch Two Sample *t* test, *p* value = 1.732 × 10^–10^). Meanwhile, there were clear non-human-infective gaps between closely related human-infective coronaviruses in the phylogenetic tree of whole genomes, and for some human-infective viruses, their closest neighbors in phylogeny were non-human-infective ones (Fig. 1c). The comparison was similar for the inferences using their spike glycoprotein coding genes (S genes) as shown in Fig. 1b (the right), and d (two-sided unpaired Welch Two Sample *t* test, *p* value = 3.657 × 10^–5^). This result is nontrivial because S genes are essential in coronavirus-host interactions^21^. Clearly, DeepHoF is good at identifying viruses infecting the same host type, whether homologous or not.

### Host prediction of SARS-CoV-2

**Figure 2:**
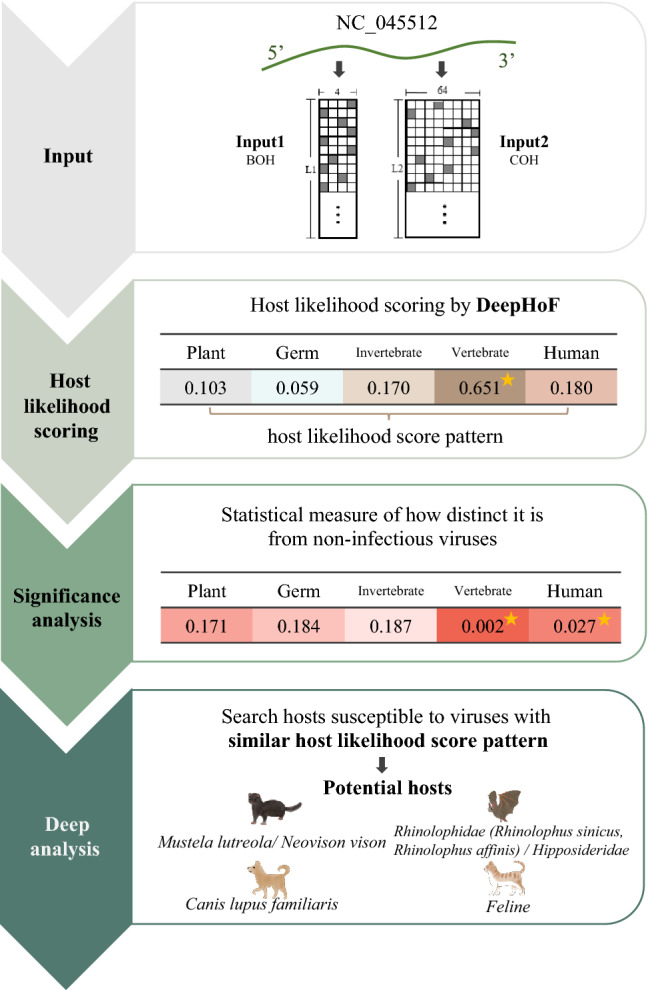
The workflow of application of DeepHoF on NC_045512. In the application of DeepHoF on SARS-CoV-2 NC_045512, the whole genome of NC_045512 was the only input required by the pre-trained DeepHoF model and coded into BOH and COH matrix for BiPathCNN network. DeepHoF output the host likelihood scores of NC_045512 on five host types respectively and the corresponding significance. The hosts of NC_045512 were predicted to be non-human vertebrates and humans with *p *values less than 0.05. Simultaneously, NC_045512 was characterized by its host likelihood score profile. Via deep analysis of host likelihood score, *Mustela lutreola/Neovison vison, Rhinolophus sinicus, Canis lupus familiaris, Hipposideros pomona, Rhinolophus affinis* and *Felidae* were output as the probable hosts of NC_045512. *BOH* base one-hot matrix, *COH* codon one-hot matrix.

The accurate prediction of hosts of the earliest detected isolates can undoubtedly assist the public health system in taking more appropriate preventive measures at the early stage of the pandemic outbreak. In view of this, we focused on the prediction with SARS-CoV-2 isolates sequenced in the earliest stage of COVID-19 detection, which is closer to the most recent common ancestor of SARS-CoV-2. We made a prediction and conducted an analysis using the 17 earliest sampled SARS-CoV-2 isolates, which provided essential information in the early epidemic of the virus. In January 2020, we reported the host predictions at the earliest stage of the outbreak using the isolates collected in December 2019^13^. Herein we used NC_045512 (complete genome of SARS-CoV-2 isolate, Wuhan-Hu-1, collected on 31 December 2019 in Wuhan, China, and used as the representative genome of SARS-CoV-2 in most studies) as an example to illustrate the workflow of DeepHoF on SARS-CoV-2 isolates (Fig. 2). The workflow of applying DeepHoF on NC_045512 is shown in Fig. 2.

**Figure 3:**
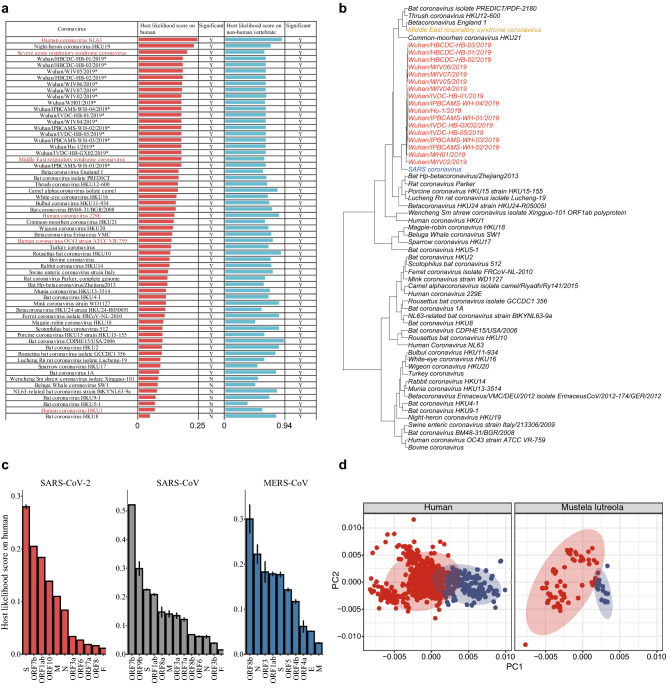
Evaluation of host likelihood scores of SARS-CoV-2. The contribution of each gene in the prediction and the visualization of host likelihood score profiles of SARS-CoV-2 isolates sampled in Netherlands. (**a**) Host likelihood scores of 17 earliest detected SARS-CoV-2 isolates and other coronaviruses on humans and non-human vertebrates. SARS-CoV-2 showed high host likelihood scores on both humans and non-human vertebrates with *p* values less than 0.05. In addition, SARS-CoV-2 was predicted lower score than SARS-CoV and comparable score to MERS-CoV on human. As for host likelihood scores on non-human vertebrates, SARS-CoV-2, SARS-CoV and MERS-CoV were close to each other. Host likelihood scores have *p* values less than 0.05 are marked ‘Y (yes)’ (red: human-infecting coronaviruses; *: the 17 earliest collected SARS-CoV-2 isolates). (**b**) Hierarchical clustering of early-stage SARS-CoV-2 and other coronaviruses using five-dimensional host likelihood score profiles given by DeepHoF. The profile of SARS-CoV-2 was close to that of SARS-CoV and MERS-CoV (red: SARS-CoV-2; blue: SARS-CoV; yellow: MERS-CoV). (**c**) Contributions of the protein coding genes on determining the host likelihood scores of SARS-CoV-2, SARS-CoV and MERS-CoV on human. The structural genes, ORF1ab and group-specific genes contributed differently in the three coronaviruses (two-sided unpaired Welch Two Sample t test, *p* value < 0.05, see in Supplementary Fig. S3). S, ORF7b and ORF1ab were the most pivotal in SARS-CoV-2. ORF7b, ORF9b and S were the most considerable in SARS-CoV. ORF8b, N and ORF3 contributed the most in MERS-CoV (S: spike glycoprotein coding gene; M: membrane/matrix glycoprotein coding gene; N: nucleocapsid phosphoprotein coding gene; E: envelope coding gene). (**d**) Principal component analysis (PCA) of host likelihood score profiles of SARS-CoV-2 detected on humans and minks in the Netherlands. All the SARS-COV-2 isolates in the Netherlands were divided into a large cluster (red) and a small cluster (blue) by the pam function of the R package cluster. The host likelihood score profiles of human-derived (left) and mink-derived (right) SARS-CoV-2 isolates in the Netherlands were distributed in a consistent mode, both containing the majority from large cluster (red) and the minority from the small cluster (blue).

For all the 17 SARS-CoV-2 isolates listed in Fig. 3a, the host likelihood scores on non-human vertebrates and humans were assigned *p* values less than 0.05 (0.002 and 0.027 respectively), illustrating a high possibility of non-human vertebrates and humans (“Materials and methods”) being hosts of SARS-CoV-2. Besides, compared to other coronaviruses released on RefSeq^22^, the high similarity of human and non-human vertebrate host likelihood scores among SARS-CoV-2, SARS-CoV and MERS-CoV (Fig. 3b), would raise an alarm when the infection capabilities of SARS-CoV-2 were uncertain in the early stage of the pandemic.

To describe the contribution of each gene in the determination of the host likelihood scores of SARS-CoV-2 isolates (using NC_045512 as a representation), we used each gene sequence of SARS-CoV-2 as the input of DeepHoF and predicted the host likelihood scores for each gene. We found that the S gene, ORF1ab and ORF7b indeed acquired high likelihood scores on the human host type and thus played important roles in determining the human as the host (Fig. 3c). The fact that several domains of the S gene and ORF1ab are essential for the coronavirus-host fusion process, host survival or viral replication^23–25^ suggests the rationality of our findings to some extent. As ORF1ab is a polyprotein that is cleaved to many non-structural proteins (nsp), we conducted a detailed analysis for ORF1ab additionally. The nucleotide sequences that code nsp 7, nsp 6 and nsp 5 were the predominant contributors on ORF1ab (Supplementary Fig. S2). It is also noteworthy that longer genes do not always have a greater influence on the prediction (Supplementary Fig. S3). Additionally, our prediction proposes the necessity of further experimental research on the function of ORF7b in SARS-CoV-2. Furthermore, we explored how each gene that functions within the coronavirus life circle^23–26^ contributed to the human host likelihood scores of SARS-CoV-2, SAR-CoV and MERS-CoV using the earliest sequenced samples, including 12 SARS-CoV isolates, 9 MERS-CoV isolates and 17 SARS-CoV-2 isolates released in NCBI in 2003, 2012 and 2019, respectively (Supplementary Table S2). The contributions of these genes were represented by their host likelihood scores on human. We found that ORF1ab was relatively important in the prediction for all these viruses, which was possibly due to its functions in viral replication and host survival^25^. Nsp 15, nsp 5 and nsp 13 were analyzed to be the most vital in ORF1ab of SARS-CoV, while nsp 1, nsp 4, and nsp 10 were the predominant in ORF1ab of MERS-CoV (Supplementary Fig. S2). The structural genes (S, M, N, and E genes) in these three viruses contributed differently to the human host type, illustrating that these genes functioned inconsistently in these viruses. Specifically, S gene, participating in the virus-host fusion process, contributed more to SARS-CoV-2 and SARS-CoV, while N gene, eliciting strong specific antibody responses, played the most important role in MERS-CoV. Two equivalent genes, ORF9b, attaching to the membrane in the virion assembly of SARS-CoV, and ORF8b, related to or immune evasion of MERS-CoV, made high contributions to human host likelihood scores for the two viruses. Moreover, two group-specific genes, ORF7b with unclear function in SARS-CoV, and ORF3 associated with viral replication and pathogenesis in MERS-CoV contributed significantly to the two viruses (Fig. 3c, Supplementary Fig. S4). These discrepancies might indicate the different significances of these genes among the three coronaviruses with regard to the interaction with human and may provide hints for targets of drug design.

It is disappointing that host determination for SARS-CoV-2 is extremely difficult due to the limited knowledge of the virus world. Therefore, the sequences and host information of viruses contained in the public database should be valued and fully utilized. To fill the gap in the efficient inference of host species for SARS-CoV-2 using the tools which were state of the art, we deeply analyzed the host likelihood profiles of viruses output by DeepHoF to seek specific vertebrate hosts of the early-stage SARS-CoV-2 isolates. In this study, we proposed that viruses with the same host species possessed host likelihood score profiles that were close in five-dimensional space. Based on this assumption, we compared the host likelihood score profile of SARS-CoV-2 with those of the non-human vertebrate viruses released in GenBank^27^ before the pandemic outbreak of SARS-CoV-2 (“Materials and methods”). We found that the host likelihood score profile of mink circovirus was the closest with that of SARS-CoV-2, followed by bat SARS-like coronavirus, canine circovirus, Pomona bat hepatitis B virus, bat coronavirus RaTG13, and feline immunodeficiency virus. It is intriguing to find that some of these viruses are phylogenetically closely related to SARS-CoV-2, while others are not, indicating that the DeepHoF algorithm does not rely on phylogenetically closely related viruses. Furthermore, according to the host label of these viruses, minks (Mustela lutreola/Neovison vison) were the most likely host, followed by Chinese rufous horseshoe bats (Rhinolophus sinicus), dogs (Canis lupus familiaris), Pomona roundleaf bats (Hipposideros Pomona), intermediate horseshoe bat (Rhinolophus affinis) and the cat family (Felidae) (Table 2, Supplementary Table S3). In contrast, minks, Chinese rufous horseshoe bats, dogs and the cat family were respectively classified into very low, low or medium groups by Damas et al.^10^, who divided 410 vertebrate species into five categories from very high to very low depending on the susceptibility to SARS-CoV-2 based on the analysis of the sequence similarity of ACE2 and the protein structure of ACE2/SARS-CoV-2 S-binding interface from the vertebrates. In the later pandemic, it should be pointed out that all the probable hosts we predicted were proven by animal experiments or the infection events^5^, which illustrated the usefulness of such an analysis for host inference of SARS-CoV-2. Remarkably, SARS-CoV-2 has been reported largely to infect farmed minks in the Netherlands, Denmark, Spain, the United States, Sweden, Italy, Greece, France, Lithuania, Canada, and Poland from April to February 2021. As of February 2021, SARS-CoV-2 had been reported to sweep 69 and 207 mink farms in the Netherlands and Denmark, respectively, which accelerated the cull of minks and destroyed the fur industry in the two countries. On 9 October 2020, at least 10,000 minks were reported dead at the Utah and Wisconsin mink farms in the USA, and they were believed to be infected by SARS-CoV-2^5^ (Table 2).

**Table 2:** Host prediction results of SARS-CoV-2.

Prediction		Evidence of infection with SARS-CoV-2^5^	Reported transmission to humans
	*Mustela lutreola/Neovison vison*	- From 19 April to 1 October, 2020, out of around 120 mink farms in Netherlands, 57 have been declared infected - From 17 June to 1 October, 2020, SARS-CoV-2 has been detected in 41 mink farms in Denmark - On 16 July, 2020, 80% of the animal samples were tested positive in a Spanish farm	- Two cases that minks transmitted SARS-CoV-2 to humans in Dutch farms were reported by Nature on 1 June 2020^11^
		- On 17 August, 2020, confirmed cases were reported in minks at two farms in Utah, the United States - On 9 October, 2020, 10,000 minks were dead at the United States fur farms and believed infected by SARS-CoV-2	
	*Rhinolophidae(Rhinolophus sinicus, Rhinolophus affinis)/Hipposideridae*	- SARS-CoV-2 is 96% identical at the whole-genome level to a bat coronavirus	N.A
	*Canis lupus familiaris*	- Confirmed cases in dogs were reported in Hong Kong, New York, Georgia, Texas, South Carolina, etc.	N.A
	*Felidae*	- Laboratory confirmed cases of cats	N.A
		- Four tigers and three lions at the same facility were all confirmed with SARS-CoV-2 in New York in April, 2020	
		- Confirmed cases in cats in New York, Minnesota, Illinois, California	

*N.A*. not available yet.

Hong Kong, Hong Kong Special Administrative Region of the People's Republic of China.

Utah, New York, Georgia, Texas, South Carolina, Minnesota, Illinois, California are states of the United States.

When evaluating the contributions of the 11 genes and the nsps on ORF1ab of SARS-CoV-2 in determining mink as the most probable host, we found that ORF1a, especially nsp 3 and nsp 5 on it, nsp 14 on ORF1b and ORF8 contributed the most (Supplementary Table S4), suggesting that genes show different contributions when determining different hosts. The rationality of this result is supported by the roles of ORF1ab in viral replication and host survival^25^, and the roles of ORF8 in immune evasion^28^. However, the interaction between the two genes and mink cells merits further attention and investigation.

Additionally, novel coronaviruses, which possess high sequence similarities with SARS-CoV-2, were found on pangolins^2,3^ in China. Although these pangolin-associated coronaviruses were assigned host likelihood score profiles similar to those of early-stage SARS-CoV-2 isolates, our analysis demonstrated that the similarity of profiles between SARS-CoV-2 and pangolin-associated coronaviruses was lower than those between SARS-CoV-2 and certain viruses of minks and Chinese rufous horseshoe bats.

### Association of SARS-CoV-2 between humans and minks

In April 2020, farmed minks in the Netherlands were noted to be infected by SARS-CoV-2 due to the abnormal mortality rate^4^. Even though all mink farms in the Netherlands have been screened mandatorily since 28 May 2020, the transmission of SARS-CoV-2 among the mink population did not seem to cease. Thus, a million farmed minks were culled in the Netherlands, followed by a plan to cull 2.5 million farmed minks in Denmark.

Based on the characterization of SARS-CoV-2 using the host likelihood score profiles, we divided all the SARS-CoV-2 isolates in the Netherlands into two clusters (a large cluster and a small cluster) via the pam function of the R package cluster. The small cluster might result from several variations in SARS-CoV-2 isolates. We then visualized the isolates detected on humans and minks individually and found the both were distributed in a consistent mode, where the majority was from the large cluster and the minority was from the small cluster (Fig. 3d, 1746 SARS-CoV-2 samples collected from humans in the Netherlands as of September 15 and 153 SARS-CoV-2 samples collected from farmed minks in the Netherlands as of October 15 were used respectively, “Materials and methods”). Consequently, the consistency hinted at the close relationship between SARS-CoV-2 isolates collected from humans and minks in the Netherlands.

This finding could be supported by the variant calling in human-derived and mink-derived SARS-CoV-2 genomes in the Netherlands. Herein we used NC_045512 as the reference, regarded variants with 5% frequency as high-frequency variants and filtered out synonymous single nucleotide polymorphisms (SNPs). Nine of 14 high-frequency variants in human-derived SARS-CoV-2 genomes sequenced in the Netherlands were absent in the genomes detected in other countries. Among these unique high-frequency variants in Dutch human-derived SARS-CoV-2, two were found in Dutch mink-derived SARS-CoV-2, thus proving the circulation of SARS-CoV-2 between humans and minks in the Netherlands (Supplementary Table S5). The associations of SARS-CoV-2 between human and mink in the Netherlands was also be supported by the conclusions from a research team in the Netherland, who utilized more detailed information about patients and related mink farms^12^. In the 2020 pandemic, minks are the only animal that has been reported to transmit SARS-CoV-2 to humans^11,12^. We further compared the high-frequency variants of SARS-CoV-2 isolates in humans and minks in the Netherlands. Except for four common variants, SARS-CoV-2 isolates derived from minks still had 23 unique high-frequency variants and six were found on the S protein, which is related to the virus-host fusion process. This result indicated that the virus might have gained a higher diversity after the intra-species circulation among mink herds and inter-species circulation between minks and human (Supplementary Table S5). As mink infections are expanding worldwide, the association and circulation of SARS-CoV-2 between humans and minks in the Netherlands notify us of the importance of taking precautions of the bidirectional transmission in other regions.

### Retrospective analysis of the pandemic

**Figure 4:**
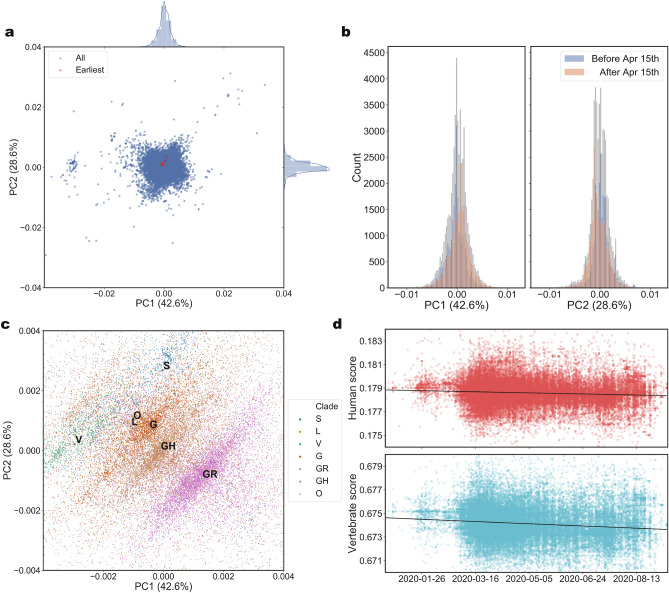
Entirety and divergence in the host likelihood score profiles of 53,759 SARS-CoV-2 isolates in the later world-wide pandemic. (**a**) PCA of host likelihood score profiles of 53,759 SARS-CoV-2 isolates and the distribution on each principal component. All the host likelihood score profiles of 53,759 SARS-CoV-2 isolates were clustered with 17 earliest sequenced isolates located in the center and the density curves displayed on each principal component were approximate normal distribution. (**b**) Distributions of host likelihood score profiles of 53,759 SARS-CoV-2 isolates collected before and after 15 April 2020. When the SARS-CoV-2 isolates were divided chronologically using 15 April 2020 as the split date, which divided the 53,759 isolates into two parts more evenly than other dates. The host likelihood score profiles of SARS-CoV-2 before and after 15 April 2020 had divergent distributions on each principal component (two-sided two-sample Kolmogorov–Smirnov test, *p* value = 0, n_isolates_ = 26,167 before 15 April 2020 and 27,592 after 15 April 2020. Blue, 26,167 isolates collected before 15 April 2020; Red, 27,592 isolates collected after 15 April 2020; Grey, all the 53,759 isolates). (**c**) GISAID clades represented in PCA of host likelihood score profiles of 53,759 SARS-CoV-2 genomes. All the 53,759 samples representing 53,759 host likelihood score profiles were painted with six different colours corresponding to six different GISAID clades of SARS-CoV-2. SARS-CoV-2 isolates fell into several clear fusiform clusters with different colours according to their clades. (**d**) Time series of the host likelihood scores on humans and non-human vertebrates for SARS-CoV-2 in the later world-wide pandemic. The host likelihood scores on humans and non-human vertebrates descend gradually with time (linear regression model analysis, *R*-squared = 6.806 $\times$ 10^–3^ and 1.431 $\times 10$^−2^, *t*_(53,757)_ =  − 19.22 and *t*_(53,757)_ =  − 27.96, *p* values = 5.543 $\times$ 10^–84^ and 3.292 $\times$ 10^–272^, slopes =  − 1.853 $\times$ 10^–6^ and − 3.768 $\times$ 10^–6^).

To verify the stability and uniformity of the host inference among SARS-CoV-2 samples, retrospective analysis of more isolates in the lasting pandemic was required. As the surge in variants of SARS-CoV-2 complicated the host prediction of the novel virus, we utilized 102,804 SARS-CoV-2 genomes available in the GISAID EpiCoV Database (https://www.gisaid.org/)^29^ as of 15 September 2020, before the rapid accumulation of mutations in SARS-CoV-2. We selected 53,759 genomes that met the quality standard given by the Chinese Academy of Sciences^30^ and trimmed their varied-length 5′- and 3′-untranslated regions (UTRs) based on the annotation of NC_045512 (Materials and methods). We calculated the host likelihood score profiles of the 53,759 isolates (Supplementary Table S5) and conducted principal component analysis (PCA) on the profiles. As shown in Fig. 4a, we found a clear cluster of all SARS-CoV-2 isolates with the 17 earliest ones located in the center. The kernel density estimation curves displayed on the first two principal components were approximately normally distributed. As the profiles of the 53,759 isolates were under the normal distribution mentioned above, the host range of SARS-CoV-2 isolates remained consistent throughout the pandemic and it is therefore reasonable that the validity of the host inference using the earliest 17 isolates would be efficient in the later pandemic.

However, when the SARS-CoV-2 isolates were divided chronologically using 15 April 2020 as the split date, which divided 53,759 isolates into two parts more evenly than other dates, we found that the two subsets had divergent distributions in each of the two dimensions of the PCA (two-sided two-sample Kolmogorov–Smirnov test, *p* value = 0, n_isolates_ = 26,167 before 15 April 2020 and 27,592 after 15 April 2020) (Fig. 4b). The approximately normal distribution of SARS-CoV-2 genomes and their time-dependent features indicate the overall consistency and a certain extent of divergence in the host likelihood score profiles of SARS-CoV-2 isolates.

To explain the divergence among host likelihood score profiles, we identified all variants in 53,759 genomes. The 13 high-frequency variants were located on the S gene, N gene, ORF1ab, ORF8 and ORF3a, some of which are related to the virus-host fusion process^21,31^ (Supplementary Table S5). Furthermore, we annotated our PCA result with the GISAID nomenclature system^29^ which divides all SARS-CoV-2 genomes into six major clades based on marker variants that appeared over time. Most of the marker variants were recognized as high-frequency variants in the variant calling (Supplementary Table S5). As shown in Fig. 4c, SARS-CoV-2 isolates fell into several clear fusiform clusters according to their clades. This indicated that those marker variants might explain the divergence among host likelihood score profiles. When we manually mutated the 17 earliest sequenced genomes with those marker variants, we found that the variants marking each clade drove the earliest sequenced SARS-CoV-2 to the corresponding cluster of the clade (Supplementary Fig. S4), which further verified our explanation of divergence. However, as the consistency of the distribution of host likelihood score profiles was not disturbed, it hinted that these mutations did not change the host range of SARS-CoV-2.

Furthermore, to explore the trend of host likelihood of SARS-CoV-2 over time, we finally examined the relationships between sampling time and the host likelihood scores on non-human vertebrates and humans (Fig. 4d). We found that both scores gradually descended. As the host likelihood scores on susceptible hosts also indicate the likelihood of being infected by SARS-CoV-2 from a computational point of view, the trends might indicate the gradually descending infectiousness to humans and other vertebrates from the outbreak to 15 September 2020. Those trends may not be so pronounced, but they should arouse our attention.

## Discussion

In summary, we proposed a deep learning method, DeepHoF, based on extracting viral genomic features, to calculate the host likelihood scores on five host types. DeepHoF made up for the vacancy of a universal tool feasible for any novel virus. For the identification of the five host types, our model can significantly outperform BLAST and discriminate between human-infective and non-human-infective viruses in a virus group such as coronaviridae. Overcoming the limitation of sequence similarity-based methods to disclose the host information of novel viruses, DeepHoF demonstrated its practicality in the prediction on SARS-CoV-2. Using 17 SARS-CoV-2 isolates sequenced in the earliest stage of COVID-19 detection, DeepHoF predicted SARS-CoV-2 could infect humans and non-human vertebrates, which had been confirmed by the pandemic. Filling the gap in predicting the host species for any novel virus that remained unsolved using the tools which were state of the art, we further analyzed the host likelihood score profile to further infer the specific hosts of SARS-CoV-2. The hosts determined by DeepHoF can be either reservoirs or susceptible middle hosts, which are not distinguished in this study. We found that mink, bat, dog and the cat family could be potential host groups of SARS-CoV-2, while minks might be one of the most noteworthy animal hosts. These top-ranking vertebrates were all naturally infected in the pandemic. Other vertebrates that have been infected naturally include gorilla and otter. In our host list, sea otter ranked 12th, and gorilla ranked 13th. Some animals, including hamster and tree shrews, which were infected experimentally received low ranking in our host list. DeepHoF could be crucial for predicting potential hosts and taking targeted action at the very beginning of an outbreak. Although we included multiple early samples in the prediction, a single early genome would suffice to apply this method. Only if sufficient adaptive changes have been accumulated in the genomes after a long enough time has elapsed since the outbreak, multiple genomes of a virus would acquire different host predictions through this approach. Actually, further analysis of all 53,759 genomes samples showed that the host likelihood score profiles of the isolates in the long period of the ensuing pandemic were slightly varied due to mutations, but followed a normal distribution where those of the early 17 isolates were located in the center. As a consequence, the host range inferred with the profiles of the isolates during the pandemic was consistent with the inference using the early samples. Additionally, based on the model, we further found that three genes (S gene, ORF7b and ORF1ab) and two genes (ORF1ab and ORF8) were significant in determining the host likelihood score on human and the host range for SARS-CoV-2, respectively. Genes involved in the virus-host fusion process (S gene), viral replication (ORF1ab) and host survival (ORF1ab) played a significant role in determining human as the host, while genes related to viral replication (ORF1ab), host survival (ORF1ab) and immune evasion (ORF8) were significant in determining the host range for SARS-CoV-2. The biological function of ORF7b is unclear now. And the mechanisms of these genes in infecting humans and other vertebrates need to be further investigated by biological experiments. For the prevention and control of a novel epidemic disease such as COVID-19, the prediction of probable hosts is essential at the early stage of the epidemic outbreak. In view of this, our study is expected to play a potentially effective role in support of those efforts.

Furthermore, according to the analysis results of host likelihood score profiles of humans and minks in the Netherlands, we found a strong association of SARS-CoV-2 isolates collected from the two populations. The analysis result of variant calling supported the finding and disclosed the contribution of mink to higher divergence in SARS-CoV-2, which could be explained by characteristics of minks in virus circulation. As reported by previous studies on avian-derived influenza A virus, minks serve as a significant node in the viral transmission network, connecting animals from different families and acting as domesticators for viral adaptation to mammals^32^. As the only one animal that has been reported to transmit SARS-CoV-2 to humans, the role of minks in the evolution of SARS-CoV-2 should be studied in depth. Therefore, with a large-scale genome analysis based on DeepHoF’s computation for the later pandemic, it should not be slighted for the relationship of SARS-CoV-2 between humans and minks.

Although we applied DeepHoF to SARS-CoV-2 in the current study, the application of DeepHoF is not limited to this virus. DeepHoF is also feasible to determine the host ranges for many other novel viruses, such as the small circular rep-encoding ssDNA viruses newly discovered on wild animals and domestic animals or in the environment. However, the limitations of DeepHoF lie in that it does not consider host sequence information, which can be improved in the future. DeepHoF also does not discriminate between reservoir hosts, vector hosts and other susceptible hosts. Meanwhile, the present study is expected to be further confirmed with both the ongoing events of the pandemic and additional experimental findings, and the interpretation of our analysis should be still kept with a certain caution.

Similar to the SARS-CoV-2 data, more complex and larger numbers of viral genome data will be produced in future epidemics. In addition, the metagenome and the metavirome can also be used in the prevention and control of the epidemic. The United States Agency for International Development launched the Global Virus Program in 2018 to reduce possible epidemiological threats by studying metaviromic samples from more than 35 countries around the world^33^. It is estimated that there are approximately 1.67 million novel viruses in mammals, birds and other important hosts of zoonotic viruses. Among them, 631,000–827,000 have the potential to cause zoonotic diseases^33^. However, only 263 viruses from 25 virus families have been confirmed to infect humans^34^. Newly emerged infectious viruses continue to threaten our health and well-being. Under these circumstances, using computational methods to discover pathogenetic viruses and acquire knowledge, including the host range, about novel viruses can provide timely responses in the prevention of epidemics and pandemics. In the future, the detection of novel viruses will rely more heavily on high-throughput sequencing technologies such as metagenomics and metaviromics. Thus, more robust tools designed for metagenomes and metaviromes are required.

## Materials and methods

### Datasets construction for training and test

We downloaded 61,684 whole viral genomes from GenBank before 9 July 2019, and tagged them with five host labels (plant, germ, invertebrate, non-human vertebrate and human), which were integrated from the host metadata provided by GenBank (Supplementary Table S6). The five host types covered all the living organism hosts. For viruses infecting multiple host types, multiple labels were given. Following the data collection procedure, short fragments were generated from tagged whole genomes because of the computational cost of long sequence processing and the varying lengths of viral genomes. The training set was constructed with short fragments from 55,283 genomes released before 1 January 2018, and the test set was constructed with the rest (the accession number, the host information and the virus lineage of the genomes used for training and test are shown in Supplementary Table S7). There is non-overlap of virus taxid in the training and test sets. The number of viral genomes for each host category and each viral taxon is summarized in Supplementary Table S8. Two groups of short fragments of two types of length ranges were generated: Group A, 100–400 bp; Group B, 400–800 bp. According to this, we built two groups of training and test sets with Group A and Group B respectively (each of the training sets contains 1,000,000 fragments, including 200,000 for each host type; each of the test sets contains 100,000 fragments, including 20,000 for each host type) (Supplementary Table S8).

### Mathematical representation of viral whole genomes

Due to the long-term adaptation to natural reservoirs, viruses share some evolutionary signatures in nucleotide sequences, such as codon pair, dinucleotide, codon, and amino acid biases, with their natural reservoirs^15^. Besides, viral proteins, especially receptors that are effectively attached to the host cell membrane, are crucial factors for viruses to invade and infect host cells^35^. In brief, the genomic compositions of viruses can inform host-virus correlation.

Herein, we represent a given viral sequence with a base one-hot matrix (BOH) and a codon one-hot matrix (COH), digitizing the genetic information of the virus at nucleotide and codon levels respectively. First, bases and codons were encoded with a one-hot format to work with deep learning algorithms. In the coding of BOH, each consecutive base of a query sequence linked by its complementary strand was encoded by one-hot. For COH, we did not extract ORFs since coding sequences make up most of the viral genome. Instead, we directly concatenated the six phases of the input sequence (Supplementary Fig. S5), and then each consecutive codon of the joined sequences was encoded by one-hot. Consequently, an input sequence of length L will be transformed to a BOH matrix with a size of 2L × 4, and a COH matrix with a size of 2L × 64.

### BiPathCNN model descriptions

In building the framework of DeepHoF, we utilized a BiPathCNN^36^, containing two CNN paths, supervised to dig information from the BOH matrix and COH matrix, respectively. This information naturally corresponds to the viral genomic features of viruses that infect the same kind of host. After independent convolution and pooling operations at the beginning, the two paths were combined by a concatenation layer. Following a normalization layer, five prediction scores were provided by five sub-paths, containing five independent nodes, corresponding to five independent binary classifications on plant, germ, invertebrate, non-human vertebrate and human individually, in the output layer with sigmoid activation and binary cross-entropy loss function for each node. The architecture of DeepHoF is shown in Supplementary Fig. S7 and the details of each layer in BiPathCNN are described in Supplementary Methods. Corresponding to the two groups of datasets, two models (model A and model B) were trained and tested. While the training and test sets in Group A are for model A, the datasets in Group B are for model B. The classification metrics for the two models were shown in Supplementary Table 9.

### Implementation of DeepHoF

In practical applications, the viral nucleotide sequence is the only input required by DeepHoF. For a viral whole genome sequence (or a partial genome sequence), a cut window moves along the long sequence without overlapping to separate it into fragments for the two pre-trained BiPathCNN models. Because the classification accuracy of model B was higher than that of model A, we gave priority to cut the input sequence into consecutive 800 bp fragments and input them into model B. For the last remaining fragment, if its length was less than 400 bp, it would be padded to 400 bp and input into model A; otherwise, it would be padded to 800 bp and input into model B. DeepHoF firstly predicted the host infection scores for each fragment. Then, it calculated the final score by weighting and summing the predicted scores of each fragment. Herein, we illustrate the separation of the input sequence and the scoring procedure with a 2000 bp sequence as input. The sequence was separated into three consecutive fragments, corresponding to the first 800 bp, the middle 800 bp and the last 400 bp of the input sequence. Then, DeepHoF predicted the three fragments independently and calculated the weighted average of the three predicted score vectors with weights of 800/2000, 800/2000, and 400/2000, respectively. For each input sequence, DeepHoF output five scores on five host types, respectively. In addition, DeepHoF provided the *p* value of each score, statistically measuring how distinct the score is compared with those of non-infectious viruses^20^. For example, if an input virus has a score of 0.4 on human, we compare 0.4 with the scores of non-human viruses in our dataset and provide the *p* value as a judgment basis. If the *p* value is less than 0.05, we conclude that human is a probable host of the input virus with a significantly higher score on the human host type than non-human viruses.

As the host likelihood score profile of a virus, consisting of the five predicted scores given by DeepHoF, can be regarded as a host-related feature vector extracted by DeepHoF, we utilized it to characterize the virus. It is logistical to regard viruses with the same host species possessing similar host likelihood score profiles. Based on this assumption, the potential host species of a virus can be inferred by the analysis of the profiles. To quantitatively compare the host likelihood score profiles between viruses, we calculated the Euclidean distance between the profiles. In the case of SARS-CoV-2, we searched the detailed non-human vertebrate host of the earliest detected isolates, which are closer to the most recent common ancestor of SARS-CoV-2. First, we added the host annotations provided by Virus-Host DB^37^ to the non-human vertebrate viruses included in GenBank. Here, the average of host likelihood score profiles of the earliest sequenced isolates was used as the representation of SARS-CoV-2. We calculated the Euclidean distance between the profile of SARS-CoV-2 and that of each non-human vertebrate virus (discovered before the outbreak of SARS-CoV-2). We regarded the vertebrate infected by a virus possessing a profile close to that of SARS-CoV-2 as the probable host of SARS-CoV-2.

### Data filtering and trimming for SARS-CoV-2 genome sequences

There were 102,804 SARS-CoV-2 genomes released on the GISAID EpiCoV Database as of 15 September 2020. We downloaded all the sequences and filtered them with the quality standard given by the Chinese Academy of Sciences^30^. Because the UTRs were not taken as seriously as the protein-coding regions and the lengths of sequenced UTRs varied greatly in different SARS-CoV-2 genomes, we trimmed the 5′- and 3′-UTRs according to the annotation of NC_045512 to eliminate noise. Thus, we finally got 53,759 clean sequences.

### Phylogenetic analysis and single nucleotide polymorphism analysis

In this study, we applied Clustal Omega software^38^ (version 1.2.4) for multiple sequence alignment and RAxML software^39^ (version 8.2.12) for phylogenetic tree building using maximum likelihood methods with 1000 bootstrap replicates. Snippy^40^ (version 4.4.3) was utilized for variant calling, using NC_045512 as the reference genome. In this study, we filtered out synonymous SNPs and regarded variants with $\ge$ 5% frequency as high-frequency variants. Commands of the three tools are included in Supplementary Information.

## Supplementary Information

Supplementary Information 1.

Supplementary Information 2.

Supplementary Information 3.

Supplementary Information 4.

Supplementary Information 5.

Supplementary Information 6.

Supplementary Information 7.

## Data Availability

Data utilized in the analysis of SARS-CoV-2, including the host likelihood score profiles and the metadata of 53,759 SARS-CoV-2 isolates, are available in the main text and Supplementary Information. The trimmed sequences of 53,759 isolates and the training and test sets of DeepHoF have been deposited on our lab homepage http://cqb.pku.edu.cn/ZhuLab/DeepHoF/.

## Code availability

The open source code utilized in this study has been deposited on GitHub https://github.com/PKUbioinfo-ZhuLab/DeepHoF and our lab homepage http://cqb.pku.edu.cn/ZhuLab/DeepHoF/.
