# Supplementary material for: Predicting hosts based on early SARS-CoV-2 samples and analyzing the 2020 pandemic
Source: Sci Rep. 2021 Aug 31;11:17422. doi: 10.1038/s41598-021-96903-6 (PMC8408148; doi:10.1038/s41598-021-96903-6)
Supplement: Supplementary file 1 — Supplementary Information 1. [file 41598_2021_96903_MOESM1_ESM.docx]

**Supplemantary Meterial for**

**Predicting hosts based on early SARS-CoV-2 samples and analyzing the 2020 pandemic**

Qian Guo^1,2,3#^, Mo Li^4#^, Chunhui Wang^4#^, Jinyuan Guo^1,3#^, Xiaoqing Jiang^1,2,5#^, Jie Tan^1^, Shufang Wu^1,2^, Peihong Wang^1^, Tingting Xiao^6^, Man Zhou^1,2^, Zhencheng Fang^1,2^, Yonghong Xiao^6*^ & Huaiqiu Zhu^1,2,3,5*^

*^1^ State Key Laboratory for Turbulence and Complex Systems, Department of Biomedical Engineering, College of Engineering, Peking University, Beijing 100871, China.*

*^2^ Center for Quantitative Biology, Peking University, Beijing 100871, China.*

*^3^ Department of Biomedical Engineering, Georgia Institute of Technology and Emory University, Georgia 30332, USA.*

*^4^ Peking University-Tsinghua University-National Institute of Biological Sciences (PTN) joint PhD program, School of Life Sciences, Peking* *University, Beijing 100871, China.*

*^5^ Institute of Medical Technology, Peking University Health Science Center, Beijing 100191, China.*

*^6^ State Key Laboratory for Diagnosis and Treatment of Infectious Diseases, National Clinical Research Center for Infectious Diseases, Collaborative Innovation Center for Diagnosis and Treatment of Infectious Diseases, The First Affiliated Hospital, College of Medicine, Zhejiang University, Hangzhou 310006, China.*

**^#^** These authors contributed equally.

^*^ Corresponding authors: hqzhu@pku.edu.cn(Zhu H) & xiao-yonghong@163.com (Xiao Y)

**The following sections include:**

Supplementary Methods

Supplementary Tables 1, 3, 6 and 9

Supplementary Figs. 1 to 7

**Other Supplementary Material for this manuscript includes the following:**

Supplementary Table S2. Metadata and host likelihood scores of genes for SARS-CoV, MERS-CoV and SARS-COV-2 isolates

### Supplementary Table S4. Contributions of 11 genes and 16 nsps in the determination of hosts for SARS-CoV-2

Supplementary Table S5. Metadata, host likelihood score profiles, and high frequency SNPs on 53759 SARS-CoV-2 isolates

Supplementary Table S7. Host information of the viral genomes in training and test sets of DeepHoF

Supplementary Table S8. The number of viral genomes for each host category and each viral taxon

Supplementary Table S10. Acknowledge of sequence data of SARS-CoV-2 in GISAID

**Supplementary Methods**

**Host prediction using BLAST**

As there are no bioinformatics tools for comparison, we compare evaluation metrics of BLAST and DeepHoF. We adopted the setting of BLAST used by Babayan et al ^15^. The host prediction using BLAST are divided into the following steps:

(1) Make BLAST database with the viral whole genomes used in the training dataset of DeepHoF. Besides, all the viral genomes are annotated with the corresponding host types.

Command: makeBLASTdb -in input_file -dbtype nucl -out database_name

(2) Align the whole genome sequences in test dataset of DeepHoF to the BLAST database made at the step (1).

Command: BLASTn -query input_file -task BLASTn -db database_name -out output_file -max_target_seqs 5 -word_size 8 -max_hsps 1 -gapopen 2 -gapextend 2 -evalue 0.001

(3) Assign host prediction result for each query sequence. For example, for query sequence A, if virus B, which is annotated with host type “human, invertebrate”, is the best hit for it, then the hosts of virus B (human and invertebrate) will be regarded as the predicted host types of viral sequence A. Meanwhile, the identity between sequence A and virus B is assigned as the prediction scores on both “human” and “invertebrate” host types, while the scores on other host types are assigned with zero. Besides, if there are no hit for viral sequence A, then the prediction scores are assigned with the difference between [1,1,1,1,1] and the true label vector. For example, if the viral A has host types of plant and germ, its label vector is [1, 1, 0, 0, 0], and if there are no hit for viral sequence A, the predicted score vector will be provided with [0, 0, 1, 1, 1] by BLAST. (Note: the five labels in the label vector are corresponding to plants, germs, invertebrates, vertebrates except human, and human, respectively)

**The details of each layer of DeepHoF**

Input: BOH(COH)

Layer b1(c1): 1D convolutional layer, including 512 convolution kernels with the length of six. Activation function: ReLU (Rectified Linear Unit, y = max(0, x)).

Layer b2(c2): global average pooling layer.

Layer 3: the concatenation layer, combining the output of the “base path” and “codon path”.

Layer 4: batch normalization layers with the dropout operation, used to facilitate the convergence and prevent the overfitting.

Layers 5a-5e, 6a-6e and 7a-7e: full connection layers, followed by batch normalization layers with the dropout operation. After the dropout operation, five sigmoid layers calculate five prediction scores, each corresponding to host likelihood scores on plants, germs, invertebrates, vertebrates and human, respectively.

**Commands of software used in phylogenetic analysis and protein annotation**

Clustalo: clustalo --full -force -i Multiple_sequence_input_file --distmat-out=name.mat --guidetree-out name.guide.nwk -o name.aln --threads 30 --outfmt=a2m

RAxML: raxmlHPC-HYBRID -s multi-alignmentfile -w outputdir -n coronavirus -m GTRGAMMA -T 40 -N 100 -p 20170808 -f a -x 20170808

Snippy: snippy --outdir outidr --ctgs fastafile --ref NC_045512.gbk --cpus 50

**Supplementary Tables**

**Supplementary** **Table S1** **Comparison of performance of DeepHoF and BLAST on each host type classification**

| Metrics | Plant | | | Germ | | Invertebrate | |
| --- | --- | --- | --- | --- | --- | --- | --- |
|  | BLAST | DeepHoF | | BLAST | DeepHoF | BLAST | DeepHoF |
| TPR | 0.725 | | 0.353 | 0.927 | 0.903 | 0.808 | 0.863 |
| FPR | 0.096 | | 0.003 | 0.234 | 0.029 | 0.130 | 0.016 |
| AUC | 0.784 | | 0.966 | 0.751 | 0.988 | 0.809 | 0.991 |
| Precision | 0.109 | | 0.643 | 0.964 | 0.995 | 0.067 | 0.377 |
| Accuracy | 0.901 | | 0.987 | 0.907 | 0.912 | 0.869 | 0.982 |
| F1-score | 0.935 | | 0.985 | 0.911 | 0.920 | 0.920 | 0.986 |

| Metrics | Vertebrate | | Human | | Average | |
| --- | --- | --- | --- | --- | --- | --- |
|  | BLAST | DeepHoF | BLAST | DeepHoF | BLAST | DeepHoF |
| TPR | 0.646 | 0.500 | 0.928 | 0.396 | 0.894 | 0.835 |
| FPR | 0.083 | 0.004 | 0.091 | 0.003 | 0.105 | 0.007 |
| AUC | 0.760 | 0.937 | 0.879 | 0.994 | 0.800 | 0.975 |
| Precision | 0.487 | 0.946 | 0.279 | 0.816 | 0.692 | 0.967 |
| Accuracy | 0.887 | 0.942 | 0.910 | 0.975 | 0.895 | 0.960 |
| F1-score | 0.894 | 0.934 | 0.932 | 0.970 | 0.899 | 0.958 |

**Supplementary** **Table S3 Top 20 hosts predicted by DeepHoF on SARS-CoV-2**

| Rank | Host name | Virus | Euclidean distance |
| --- | --- | --- | --- |
| 1 | Neovison vison/ Mustela lutreola | Mink circovirus | 0.019 |
| 2 | Rhinolophus sinicus | Bat SARS-like coronavirus | 0.026 |
| 3 | Canis lupus familiaris | Canine circovirus | 0.035 |
| 4 | Hipposideros pomona | Pomona bat hepatitis B virus | 0.039 |
| 5 | Rhinolophus affinis | Bat coronavirus RaTG13 | 0.043 |
| 6 | Feliformia/ Felidae | Feline immunodeficiency virus | 0.051 |
| 7 | Ailuropoda melanoleuca | Giant panda anellovirus | 0.055 |
| 8 | Meleagris gallopavo | Turkey coronavirus | 0.066 |
| 9 | Paguma larvata | SARS coronavirus | 0.072 |
| 10 | Sus scrofa | Porcine parvovirus 5 | 0.073 |
| 11 | Plecotus auritus | Bat associated circovirus 5 | 0.074 |
| 12 | Enhydra lutris kenyoni | Sea otter poxvirus | 0.074 |
| 13 | Gorilla gorilla | Bocavirus gorilla/GBoV1/2009 | 0.078 |
| 14 | Galliformes/ Gallus gallus | Reticuloendotheliosis virus | 0.079 |
| 15 | Canis lupus signatus | Lupine bocavirus | 0.081 |
| 16 | Pan troglodytes | Simian foamy virus | 0.082 |
| 17 | Gallus gallus | Avian infectious bronchitis virus | 0.086 |
| 18 | Pipistrellus | Bat coronavirus isolate PREDICT/PDF-2180 | 0.087 |
| 19 | Chelonia mydas | Sea Turtle Tornovirus | 0.090 |
| 20 | Zosteropidae | White-eye coronavirus | 0.091 |

**Supplementary** **Table S6 Subtypes in five host types**

| Host type | Host subtypes |
| --- | --- |
|  |  |
|  |  |
| plant | algae, diatom, plant |
| germ | Archaea, bacteria, fungi, protozoa |
| invertebrate | invertebrate |
| vertebrate | vertebrates except human |
| human | human |

**Supplementary Table S9. The classification metrics for (for 100-400bp fragments) and model B (for 400-800bp fragments).**

| Metrics | Model A (for 100-400bp fragments) | | | | | |
| --- | --- | --- | --- | --- | --- | --- |
|  | Precision | Accuracy | TPR | FPR | AUC | F1-score |
| Plant | 0.673 | 0.822 | 0.231 | 0.028 | 0.860 | 0.786 |
| Germ | 0.868 | 0.902 | 0.701 | 0.034 | 0.948 | 0.899 |
| Invertebrate | 0.744 | 0.868 | 0.745 | 0.090 | 0.924 | 0.868 |
| Vertebrate | 0.912 | 0.845 | 0.769 | 0.077 | 0.926 | 0.844 |
| Human | 0.869 | 0.944 | 0.768 | 0.022 | 0.972 | 0.942 |
| Average | 0.844 | 0.876 | 0.674 | 0.047 | 0.926 | 0.872 |

| Metrics | Model B (for 400-800bp fragments) | | | | | |
| --- | --- | --- | --- | --- | --- | --- |
|  | Precision | Accuracy | TPR | FPR | AUC | F1-score |
| Plant | 0.901 | 0.879 | 0.448 | 0.012 | 0.957 | 0.862 |
| Germ | 0.954 | 0.960 | 0.877 | 0.014 | 0.990 | 0.959 |
| Invertebrate | 0.834 | 0.938 | 0.948 | 0.066 | 0.987 | 0.939 |
| Vertebrate | 0.951 | 0.948 | 0.946 | 0.051 | 0.987 | 0.948 |
| Human | 0.864 | 0.967 | 0.935 | 0.027 | 0.992 | 0.968 |
| Average | 0.910 | 0.938 | 0.860 | 0.032 | 0.983 | 0.938 |

**Supplementary Figures**

**
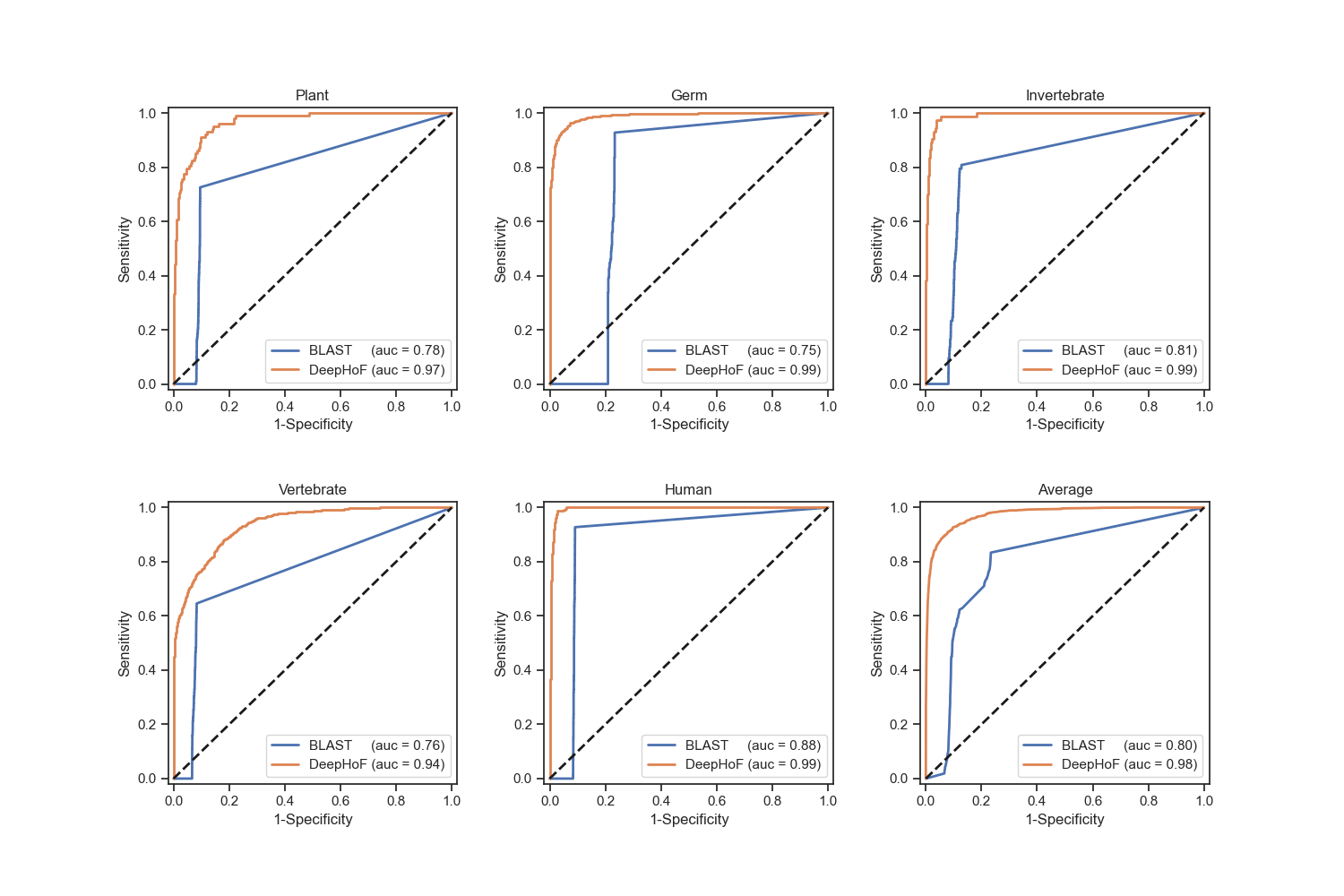
**

**Supplementary** **Fig. 1 ROC curves and AUC values of DeepHoF and BLAST on five host types**

DeepHoF performs better than BLAST on AUC of each host type.

**Supplementary** **Fig. S2 Contributions of the nsps on determining the human host likelihood scores of SARS-CoV-2, SARS-CoV and MERS-CoV.**

Left, for SARS-CoV-2, nsp 7, nsp 6 and nsp 5 were the predominant contributors on ORF1ab. Middle, for SARS-CoV, nsp 15, nsp 5 and nsp 13 were the predominant contributors on ORF1ab. Right, for MERS-CoV, nsp 1, nsp 4, and nsp 10 were the predominant contributors on ORF1ab.

**Supplementary** **Fig. S3** **The scatter plot showing the host likelihood scores of SARS-CoV-2 genes with varying lengths.**

For the genes of SARS-CoV-2, the longer genes do not always have a greater influence on the host likelihood scores on plant (**A**), germ (**B**), invertebrate (**C**), vertebrate (**D**) and human (**E**).


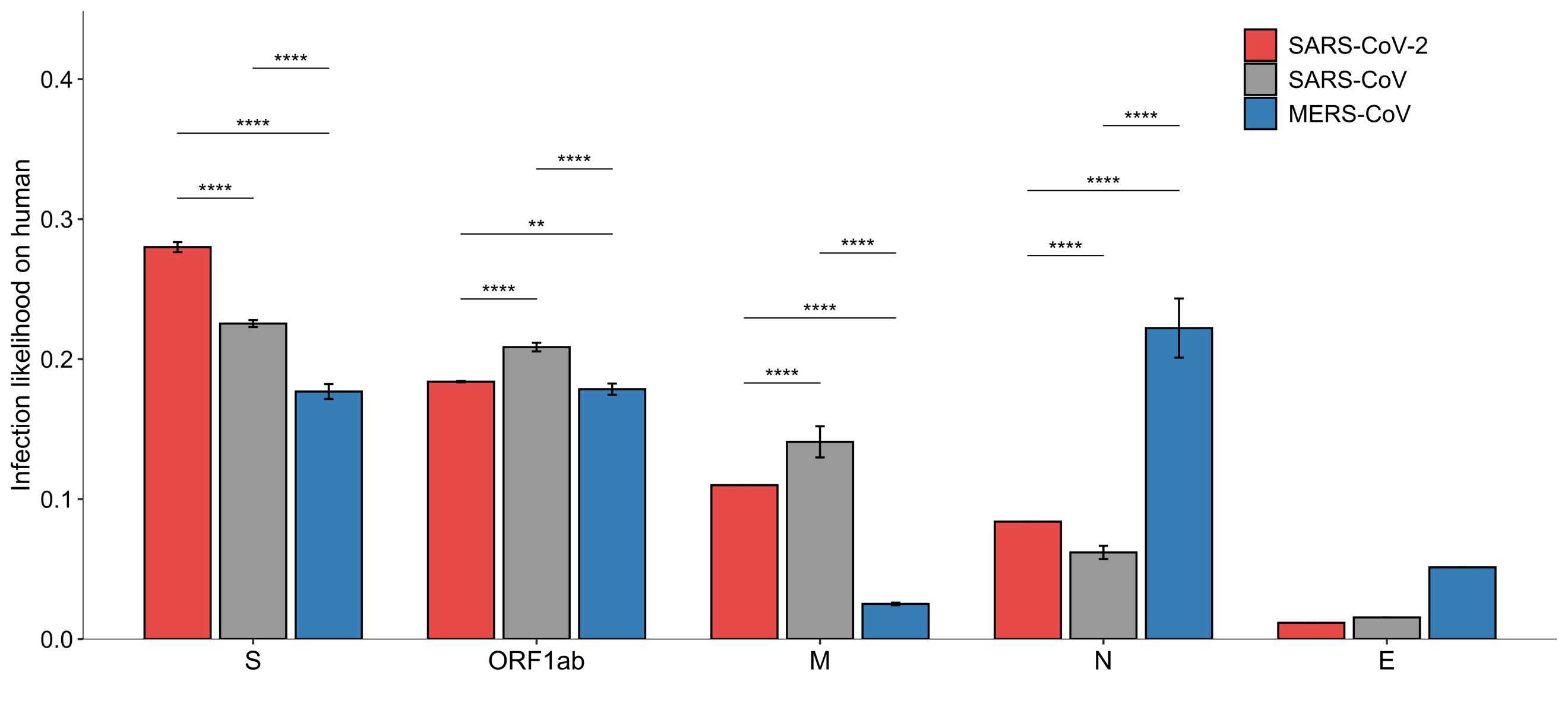


**Supplementary** **Fig. S4 Human host likelihood scores of 5 genes of SARS-CoV-2, SARS-CoV and MERS-CoV**

Although all the three coronaviruses possess ORF1ab and four structural genes (S, M, N, E), these genes made different contributions on human host likelihood scores in these three viruses (two-sided unpaired Welch Two Sample *t*-test, *p*-value $<$ 0.05). S gene and M gene contributed more in SARS-CoV-2 and SARS-CoV, while N gene and E gene were more significant in MERS-CoV.


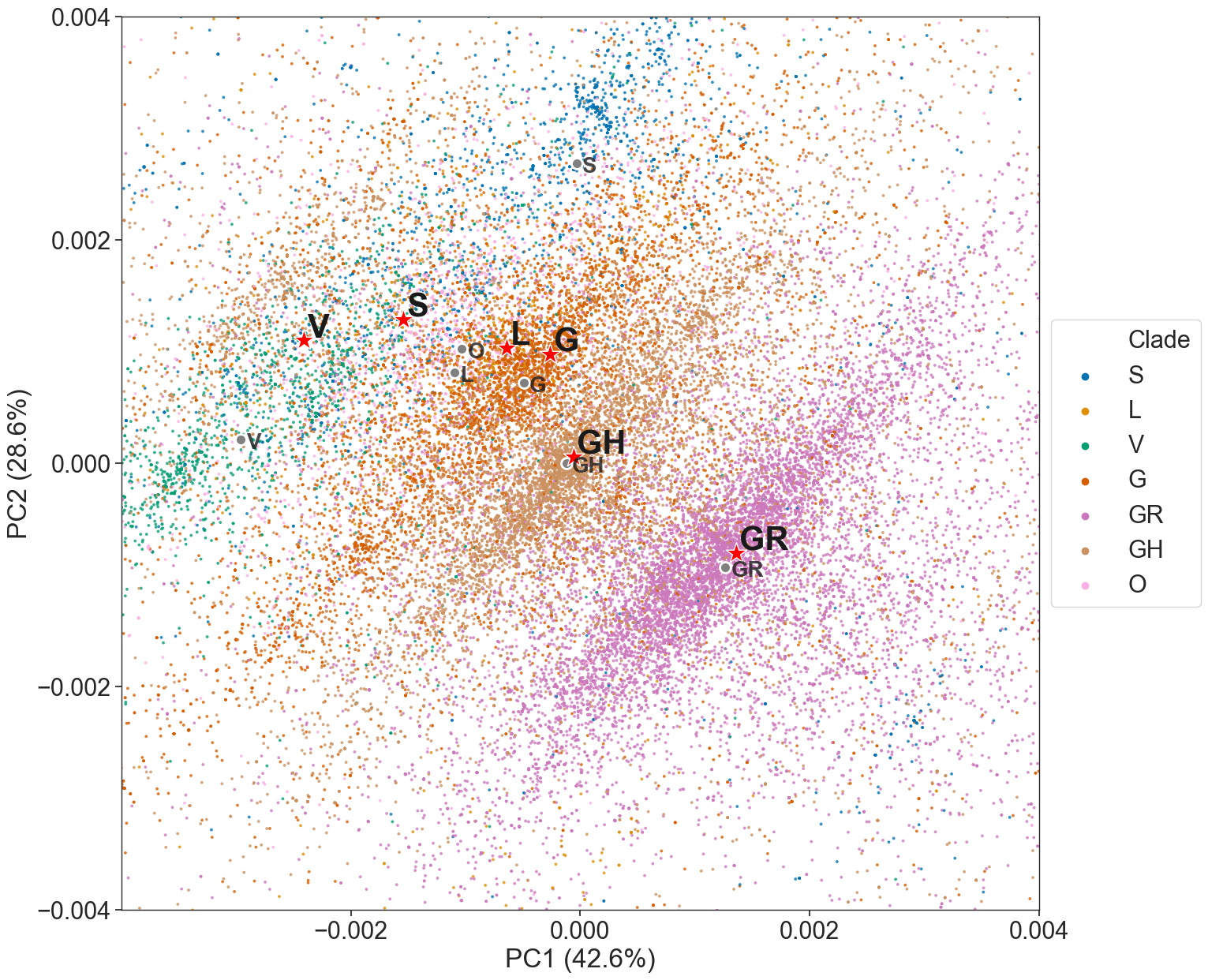


**Supplementary** **Fig. S5 Visualization of the host likelihood** **score profiles of SARS-CoV-2 isolates from different GISAID clades and the manually mutated SARS-CoV-2 isolates on two-dimensional PCA**

SARS-CoV-2 isolates fall into several clear fusiform clusters with different colors according to their clades. Manually mutated with specific marker variants, the 17 earliest sequenced isolates move to the corresponding fusiform cluster of the clade that is represented by the specific marker variants.


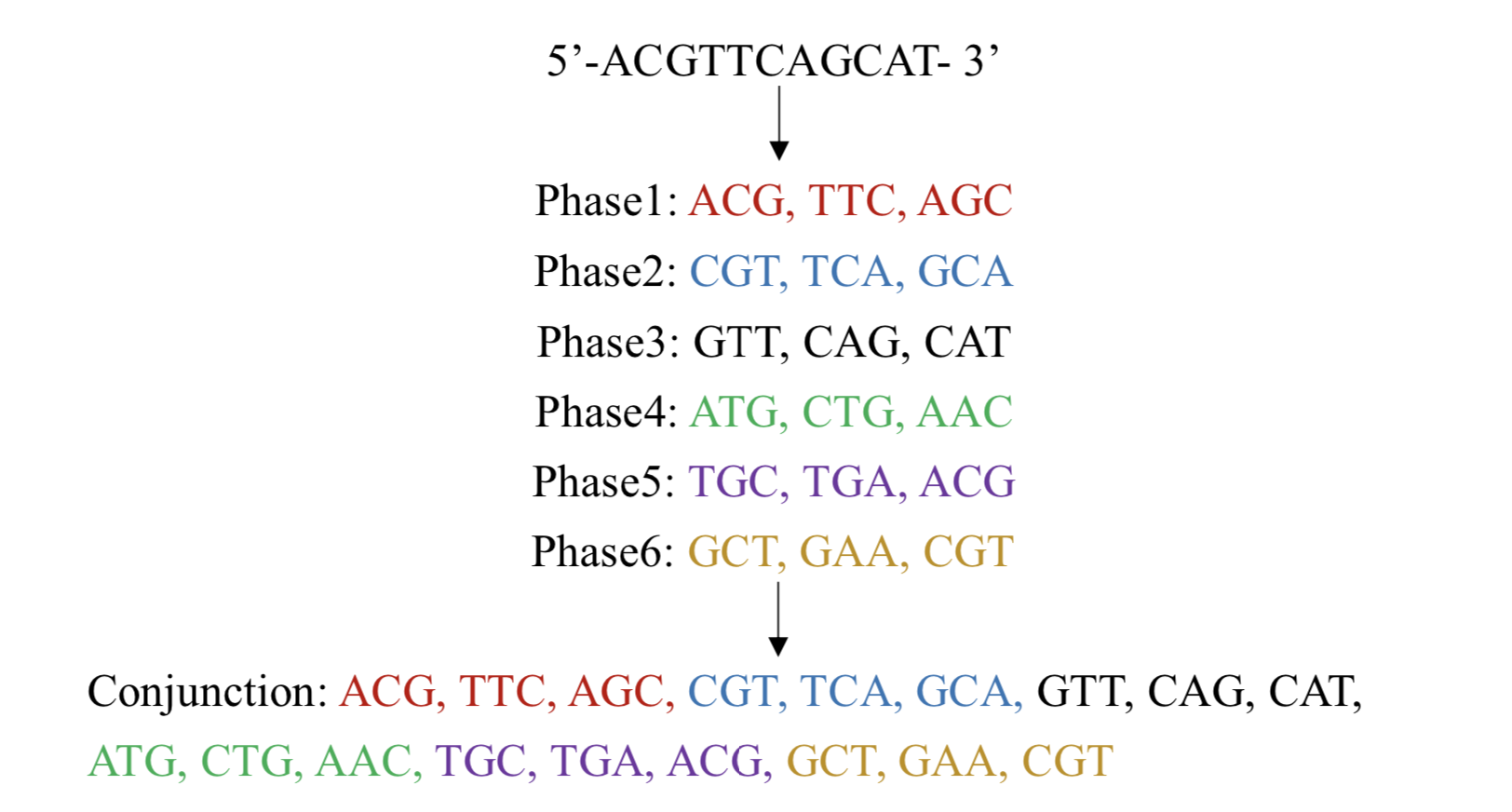


**Supplementary** **Fig. S6 Six phases of an input sequence.**

For coding the COH matrix of a given sequence, we represented it with the direct conjunction of its six phases, generated from its complementary strand and itself.


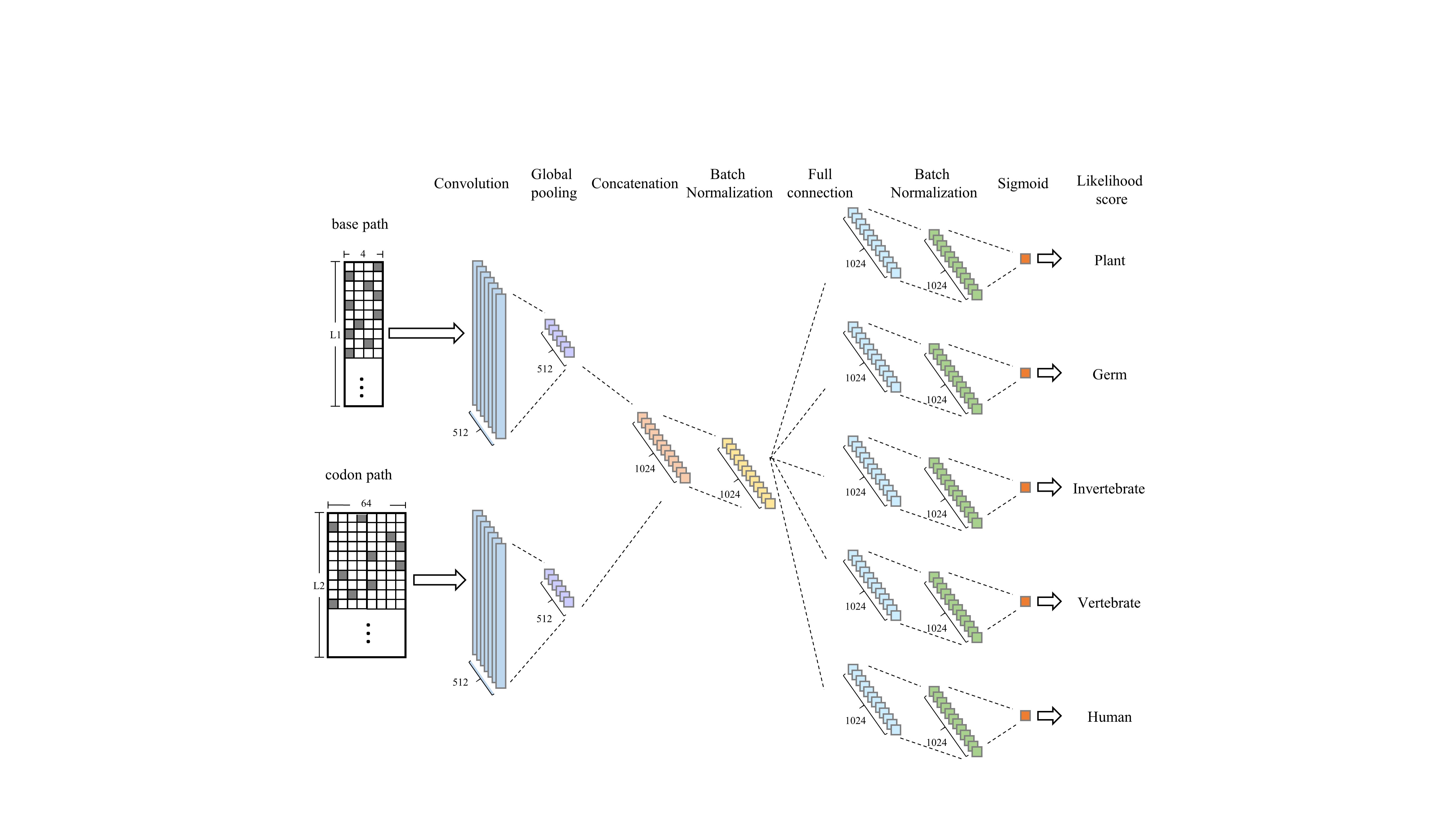


**Supplementary** **Fig. S7 Structure of BiPathCNN in DeepHoF**

BOH matrix and COH matrix are input into two paths independently and transformed by the convolution and pooling layers at the beginning. A concatenation layer and a normalization layer combine the output of the two paths. Five sub-paths process the combined intermediate output individually. Each sub-path contains a full connection layer, a normalization layer and an output layer with sigmoid activation and binary cross-entropy loss function. The five sub-paths output the host likelihood scores on five host types respectively.
